# Transcatheter Tricuspid Valve-in-Valve Replacement Using a J-Valve System for a Failed Tricuspid Bioprosthesis

**DOI:** 10.1155/2022/7353522

**Published:** 2022-06-29

**Authors:** Mingkui Zhang, Hui Xue, Lifu Miao, Xiujie Tang, Yanbin Shao

**Affiliations:** Heart Center, First Hospital of Tsinghua University, No. 6 1st Street, Jiuxianqiao, Chaoyang District, Beijing 100016, China

## Abstract

**Background:**

Redo operation for failed tricuspid bioprosthetic valves is associated with high morbidity and mortality. Transcatheter tricuspid valve-in-valve implantation has become an acceptable option for high-risk patients with a failed tricuspid bioprosthesis. We present a case of successful tricuspid valve-in-valve implantation using a J-valve in a failed tricuspid bioprosthesis position. *Case Summary*. A 48-year-old male, who had a failed tricuspid bioprosthesis, presented with right-side heart failure, right-to-left shunting at the atrial level, severe dyspnea, cyanosis, peripheral edema, hepatauxe, and ascites. After the interdisciplinary assessment, we successfully performed transcatheter tricuspid valve-in-valve implantation with the J-valve system. At 34-month postoperative follow-up, the patient had no symptoms of heart failure and the echocardiogram showed good valve position and well hemodynamic status.

**Conclusions:**

This case demonstrated that the J-valve system may be a new option for high-risk patients with a failed tricuspid bioprosthetic valve.

## 1. Introduction

The bioprosthetic valve has become the main choice for tricuspid valve replacement because of its advantages such as low incidence of thrombosis and avoidance of complications associated with anticoagulation [1, 2]. However, bioprosthetic valve failure is inevitable in the long term due to degeneration and calcification [3]. Reoperation is the standard treatment for failed bioprosthetic valves, but it may carry significant risks of adverse perioperative events [4]. The first-in-man tricuspid valve-in-valve (VIV) was reported in 2011 using the off-label Edwards SAPIEN valve (Edwards Lifesciences). Since then, transcatheter tricuspid VIV implantation has become an acceptable alternative for high-risk patients with a failed tricuspid bioprosthesis [5–8]. The J-valve system (Jiecheng Medical Technology Co., Ltd., Suzhou China) is a new second generation of transcatheter heart valves and has been an adequate option for the treatment of high-risk patients with aortic valve stenosis and regurgitation [9]. We describe a man with severe bioprosthetic tricuspid valve (TV) stenosis who underwent transcatheter tricuspid valve-in-valve with the use of a J-valve system.

## 2. Case Presentation

In August 2019, a 48-year-old male presented at our hospital due to severe dyspnea (NYHA III), cyanosis, dizziness, peripheral edema, hepatauxe, and massive ascites. He had a history of the congenital ventricular septal defect, infective endocarditis, heart valve infective vegetation, and severe tricuspid valve regurgitation and undergone ventricular septal defect repair, aortic valve repair, pulmonary valve repair, and the tricuspid valve replacement with a 29 mm bioprosthesis (Edwards Lifesciences, LLC; Irvine, California) in another hospital in 2005. Severe postoperative pulmonary infection complications occurred and required tracheotomy and ventilator-assisted therapy for more than seven days. From the beginning of 2017, he gradually started to present heart failure symptoms and underwent multiple hospital admissions for severe dyspnea, peripheral edema, and recurrent ascites. The current physical examination revealed lethargy, cyanotic lips, jugular vein distension, a grade of 2/6 diastolic murmur at the left lower sternal border, hepatauxe, and massive ascites. Blood gas analysis shows that oxygen pressure is 56.8 mmHg, oxygen saturation is 88.2%, carbon dioxide is 27.6 mmHg, and hemoglobin is 231 g/L.

**Figure 1:**
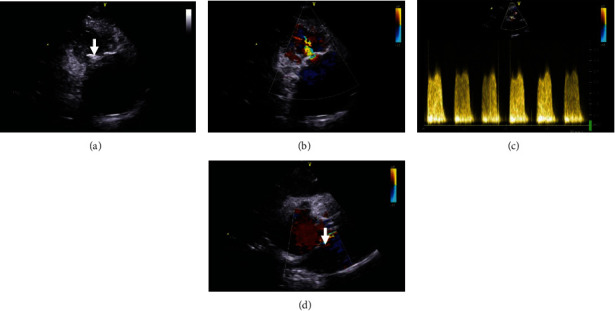
Transthoracic echocardiography (TTE) and Doppler before the operation: bioprosthetic tricuspid valve calcification (white arrow) and stenosis (a, b). Bioprosthetic maximal velocity 2.7 m/s (c). The foramen ovale opening (white arrow) (d).

**Figure 2:**
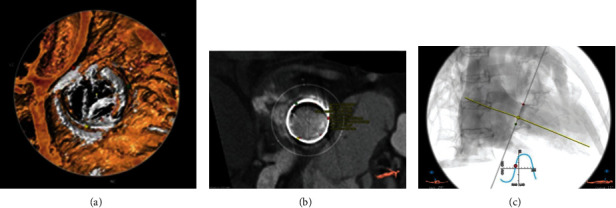
Cardiac computed tomography images: distribution of severe bioprosthetic leaflet calcification (a). Inner diameter of the bioprosthetic valve (26.3 mm) (b). The best projection angles (c).

Transthoracic echocardiography revealed bioprosthetic TV stenosis (Figure 1). The mean transvalvular gradient pressure and peak transvalvular gradient pressure were 21 mmHg and 33 mmHg, respectively. The aortic valve had mild regurgitation, foramen ovale opening (6 mm), and right-to-left shunting. The right atrial and ventricular volumes were enlarged. The tricuspid annular plane systolic excursion was 8 mm, and the fractional area change (FAC) was 40%. The distribution of severe calcification in the bioprosthetic valve and the bioprosthesis annulus's diameter were determined by multidetector computed tomography (MDCT) (Figure 2). Diagnostic cardiac catheterization examination showed that the superior vena cava pressure, the inferior vena cava pressure, and the right atrial pressure were 29/12/19 mmHg, 28/20/23 mmHg, and 29/16/21 mmHg, respectively. These findings were consistent with a severe stenosis of the bioprosthesis in the tricuspid position.

After the interdisciplinary assessment, the heart team decided to perform transcatheter tricuspid VIV implantation using the J-valve system because of a significantly increased surgical risk for conventional redo surgery: the estimated surgical risks (EuroSCORE II) were 12.4%. Based on MDCT measurements, the 27 mm J-valve was suitable for valve-in-valve replacement in the tricuspid position.

**Figure 3:**
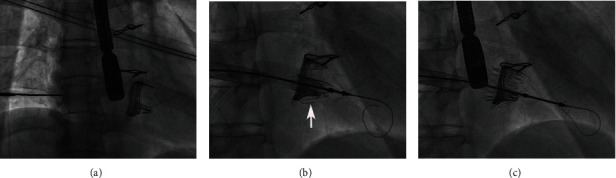
Step-by-step transatrial TVIV implantation of the J-valve. The right atrium puncture according to the coaxial position of the tricuspid annulus (a). The three “U-shape graspers” were released and embraced the prior bioprosthetic struts (white arrow) (b). Full deployment of the J-valve (c).

After confirmed consent and Hospital Ethics Committee approval (no. 20190009) were obtained, the procedure was performed in a hybrid operating room under general anesthesia with a double-lumen endotracheal tube. Transesophageal echocardiogram (TEE) was used for the evaluation of the valve pathology and operation result. Both the right femoral artery and vein were exposed for emergency cardiopulmonary bypass. The left radial artery and right internal jugular vein puncture catheterization was performed for measurement of blood pressure and central venous pressure. A right minithoracotomy in the fourth intercostal space and the right atrial double purse-string sutures were performed according to the coaxial position of the tricuspid annulus. Heparin was administered to keep the activated clotting time at more than 300 seconds. After the right atrium puncture, a soft guide wire and then a superstiff guide wire were used to cross the bioprosthetic valve and into the right ventricle. The valvuloplasty (balloon inflated to 22 mm, Percutaneous Transluminal Valvuloplasty Catheter Z-MED II™, NuMED, CAN) was performed without rapid ventricular pacing. The J-valve was reversely loaded on the conveyor system and successfully implanted into the degenerated bioprosthesis. The three “U-shape graspers” were released and embraced the prior bioprosthetic struts (Figure 3). Intraoperative transesophageal echocardiograms confirmed the correct position, no periprosthetic leakage, right-to-left intracardiac shunt vanishment at the atrial level, and excellent function of the tricuspid bioprosthetic valve, with maximal pressure gradient 12 mmHg. Artery oxygen saturation and hemodynamic status were improved shortly.

**Figure 4:**
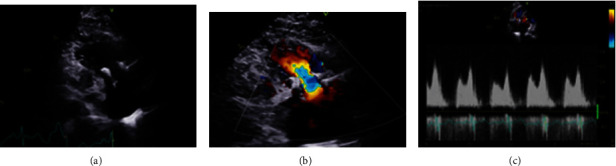
The transthoracic echocardiogram images during the 34-month follow-up period: TTE showed optimal valve position and well hemodynamic status (a, b). TTE with color flow showed a maximal velocity of 1.6 m/s (c).

Postoperatively, oral warfarin anticoagulation was used to maintain an internationalization ratio of 2.0-2.5 for three months. Transthoracic echocardiography and electrocardiogram were performed regularly for postoperative evaluation. Thirty-four months after surgery, the patient was free of heart failure symptoms and the echocardiogram suggested excellent hemodynamics of the bioprosthetic valve (Figure 4).

## 3. Discussion

Transcatheter VIV implantation has become an attractive option for failed aortic and mitral bioprostheses. The American Food and Drug Administration approved the mitral VIV procedure with the Edwards SAPIEN system in 2017 [10]. Recent publications reported good results following implanted stent valves in the aortic or mitral position [11, 12]. Since the first case of tricuspid valve-in-valve using an off-label Edwards SAPIEN valve (Edwards Lifesciences Corporation, Irvine, CA, USA) was reported, the literature on VIV implantation in failed tricuspid bioprostheses is reported in small case series and case reports [5, 13, 14]. Recently, registry data was reported by McElhinney, demonstrating that transcatheter tricuspid VIV implantation was hemodynamically and clinically beneficial in patients of various ages and underlying disease states. The adverse TVIV-related outcomes (TV dysfunction, endocarditis, and leaflet thrombosis) were relatively uncommon in 306 patients, and valve function remained excellent in the majority of patients in three-year follow-up results [15].

The J-valve is a new generation self-expendable valve and has previously been shown to be effective for the treatment of both serve aortic valve stenosis and aortic regurgitation. A J-valve multicenter study, which enrolled 107 high-risk patients with aortic valve stenosis, aortic valve regurgitation, or bicuspid aortic valve (BAV), demonstrated a lower rate of complications and mortality at 1-year follow-up [16]. The J-valve has several advantages: firstly, it is a short stent-valve frame that avoids the right ventricular outflow tract obstruction. Secondly, the unique 3 U-shaped graspers facilitate anchoring and avoid displacement after deployment. Thirdly, the shortest distance through the right atrium access facilitates the coaxiality of the implanted valve and is easy to manipulate. For this patient, the off-label procedure was the last resort to improve his quality of life, and the decision was made after a full discussion between the patient and the heart team. At the 34-month postoperative follow-up, the patient had no symptoms of heart failure and the echocardiogram showed good valve hemodynamics.

## 4. Conclusion

Transcatheter tricuspid VIV implantation has become an acceptable alternative to conventional open surgery for patients with a failed tricuspid bioprosthesis. This case demonstrated that transcatheter VIV replacement using a J-valve system may be a new option for the failed tricuspid bioprosthesis patients.

## Data Availability

The data generated or analyzed during this study in this published article are available from the corresponding author on reasonable request.
